# Chronic Granulomatous Disease: A Large, Single-center US Experience

**DOI:** 10.1097/INF.0000000000000840

**Published:** 2015-09-15

**Authors:** Pietro Bortoletto, Kyle Lyman, Andres Camacho, Marielle Fricchione, Aaruni Khanolkar, Ben Z. Katz

**Affiliations:** From the * Department of Pediatrics, Northwestern University Feinberg School of Medicine; † Ann & Robert H. Lurie Children’s Hospital of Chicago; and ‡ Department of Pathology, Northwestern University Feinberg School of Medicine, Chicago, Illinois.

**Keywords:** chronic granulomatous disease, interferon-outcomes, antimicrobial prophylaxis, x-linked, autosomal recessive

## Abstract

**Background:**

Chronic granulomatous disease (CGD) is an uncommon primary immunodeficiency that can be inherited in an X-linked (XL) or an autosomal recessive (AR) manner. We reviewed our large, single-center US experience with CGD.

**Methods:**

We reviewed 27 patients at Ann & Robert H. Lurie Children’s Hospital of Chicago from March 1985 to November 2013. Fisher exact test was used to compare differences in categorical variables, and Student *t* test was used to compare means for continuous variables. Serious infections were defined as those requiring intravenous antibiotics or hospitalization.

**Results:**

There were 23 males and 4 females; 19 were XL and 8 were AR. The average age at diagnosis was 3.0 years; 2.1 years for XL and 5.3 years for AR inheritance (*P* = 0.02). There were 128 serious infections. The most frequent infectious agents were *Staphylococcus aureus* (n = 13), *Serratia* (n = 11), *Klebsiella* (n = 7), *Aspergillus* (n = 6) and *Burkholderia* (n = 4). The most common serious infections were pneumonia (n = 38), abscess (n = 32) and lymphadenitis (n = 29). Thirteen patients had granulomatous complications. Five patients were below the 5th percentile for height and 4 were below the 5th percentile for weight. Average length of follow-up after diagnosis was 10.1 years. Twenty-four patients were compliant and maintained on interferon-γ, trimethoprim-sulfamethoxazole and an azole. The serious infection rate was 0.62 per patient-year. Twenty-three patients are alive (1 was lost to follow-up).

**Conclusions:**

We present a large, single-center US experience with CGD. Twenty-three of 27 patients are alive after 3276 patient-months of follow-up (1 has been lost to follow-up), and our serious infection rate was 0.62 per patient-year.

Chronic granulomatous disease (CGD) was first described in 1957. By 1966, it was understood that this disease was because of an inability of polymorphonuclear leukocytes to kill ingested bacteria and it was believed to be exclusively X-linked (XL); the diagnostic nitroblue tetrazolium test was first described a year later (reviewed in Ref. ^1^). The first cases of what were postulated to be autosomal recessive (AR) CGD were described a year after that in 1968.^2^ The disease is estimated to occur in approximately 1 of 100,000 to 1 of 200,000 births,^3–9^ with the incidence differing among ethnic groups.^8^ Patients with CGD have defects in nicotinamide adenine dinucleotide phosphate (NADPH) oxidase, which has membrane-associated (eg, gp91-phox and p22-phox) and cytosolic components (eg, p47-phox and p67-phox). The gp91-phox defect is inherited in an XL manner, whereas the p22-phox, p47-phox and p67-phox defects are inherited in an AR manner. The XL form is more severe, presents earlier and has a higher mortality.^3,4,10–12^ Because of the defects in NADPH oxidase, phagocytes from patients with CGD are able to ingest pathogens but are unable to mount a respiratory burst of hydrogen peroxide and kill them. Catalase-positive bacteria and fungi such as staphylococci, *Serratia*, *Burkholderia*, and *Aspergillus* are particularly important pathogens in patients with CGD, because they express some catalase and thus can neutralize the small amounts of hydrogen peroxide that phagocytes produce by mechanisms other than NADPH oxidase.^3,4,10–12^

The most common types of infections seen in patients with CGD are pneumonia, lymphadenitis and abscesses; among the most common infectious organisms reported are *Staphylococcus aureus* and *Aspergillus*. As with many immune deficiencies, immunologic dysregulation can also be seen, the most common syndromes of which are colitis and obstructive granulomatous lesions.^3,4,10–12^ Mean heights and weights can be reduced in patients with CGD.^5,11,13,14^

Morbidity has been reported as 0.26–1.1 severe infections per patient-year,^6,10–12,15^ with 1 study in the mid-1990s reporting 1.7 severe infections per patient-year in those with the XL form and 0.5 severe infections per patient-year in those with the AR form.^6^ Death is often because of *Aspergillus* or *Burkholderia* infection.^3,4,10–12^

Mortality in patients with CGD has steadily declined over time. The first review of this disease, published in 1967, described more than 50% mortality by age 6 and almost 65% mortality by age 7.^1^ A study published in 1990 reported 30% mortality by age 10 and 50% by age 20.^13^ Another study published at around that time documented improved survival with time, by showing that children born before 1978 had an 8-year survival of approximately 70%, whereas children born after 1978 had a better than 90% chance of surviving to age 8.^16^ In studies published since 2000, mortality has been generally shown to be higher for patients with the XL form than those with the AR form,^3,4^ with 1 recent study reporting a median survival of almost 50% 25 years after diagnosis.^12^

Treatment often consists of antibacterial prophylaxis with trimethoprim-sulfamethoxazole, antifungal prophylaxis with an azole (usually itraconazole^17^) and immunotherapy with interferon-γ, all of which have been shown to reduce infections in CGD,^17–20^ the latter 2 in randomized, double-blind, placebo-controlled trials and the former based on retrospective analyses. Gene therapy (reviewed in Ref. ^21^) and bone marrow or stem-cell transplantation^4,8,9,11,12,22–27^ hold out the promise of cure. The objective of our analysis was to review our large, single-center US experience with CGD.

## MATERIAL AND METHODS

This was a retrospective review of all 27 patients with CGD followed up at the Ann & Robert H. Lurie Children’s Hospital of Chicago (formerly Children’s Memorial Hospital) from March 1985 to November 2013. All available paper clinic charts, paper hospital charts and electronic medical records were searched. This study was approved by the Institutional Review Board of the Children’s Research Center. Some of these patients have been referred to in previous publications.^28–30^

Diagnosis was established by quantifying the patient’s neutrophil oxidative burst. Earlier patients were studied with the nitroblue tetrazolium test, later patients (after the mid-1990s) were studied through flow cytometry.^31^ Genotyping was performed [courtesy of the Scripps Research Institute (La Jolla, CA) or the National Institutes of Health (Washington, DC)].

Serious infections were defined as those related to CGD that required parenteral antibiotics and/or inpatient hospitalization.^10–12^ We did not count infection-related admissions not attributable to the CGD immune-deficient state, eg, a urinary tract infection in a neonate (before the diagnosis of CGD was established) in 1 patient or dehydration secondary to rotavirus unassociated with colitis in another. Charts were reviewed by at least 2 physicians who agreed with these assignments in every case. Some infections (eg, liver abscesses) required months of intravenous therapy and multiple admissions; however, each of these prolonged episodes of infection was counted as a single infection.

Fisher exact test was used to compare differences in categorical variables, and Student *t* test was used to compare means for continuous variables. All *P* values are 2-sided and considered significant when *P* < 0.05. Months of survival were described by Kaplan–Meier estimates.

## RESULTS

### Demographics

Our cohort consisted of 23 males and 4 females, including 4 groups of siblings that accounted for 11 patients. Two groups of siblings were from consanguineous marriages: 1 pair of female siblings and a group of 3 brothers (one of whom was severely affected and 2 of whom remain asymptomatic). Of those who identified an ethnicity/nationality, 8 were Caucasian (including 2 who were identified as Polish and 2 who were identified as Serbian), 9 were Hispanic, 5 were Middle Eastern and 1 was Black. Ten of the patients had private insurance; the rest were Medicaid.

Ages ranged from 1 to 29 years at the time of follow-up. Three patients were born between 1980 and 1984, 1 was born between 1985 and 1989, 7 were born between 1990 and 1994, 3 were born between 1995 and 1999, 4 were born between 2000 and 2004, 6 were born between 2005 and 2009 and 3 were born between 2010 and November 2013. The mean age at diagnosis was 4.9 years for patients born between 1990 and 1994, 6.6 years for those born between 1995 and 1999, 2.9 years for those born between 2000 and 2004, 1.4 years for those born between 2004 and 2009 and 0.6 years for those born between 2010 and November 2013. Of the 7 patients aged 18 years or older at the time they were last seen, 3 lived at home, 2 were attending college (one was a sophomore and the other a senior) away from home and 2 lived independently. The college senior had several job offers for the following year. None were married. They are all still followed up in our Clinic, although we are in the process of transferring our oldest patient to an adult practitioner.

### Genetics

Nineteen of our patients had an XL mode of inheritance and 8 had an AR (*P* = 0.034) mode. The average age at diagnosis was 3.0 years; 2.1 years for those with XL inheritance and 5.3 years for those with AR inheritance (*P* = 0.02). Of the 11 patients who were genotyped, 7 had defects in gp91-phox (XL) and 2 each had defects in p47-phox or p67-phox (AR).

### Follow-up

There were a total of 3276 person-months of follow-up. Thus, the average length of follow-up after diagnosis was 10.1 years, 9.8 years in the patients with XL inheritance and 10.6 years in those with AR inheritance.

### Clinical Manifestations

There were 128 serious infections, 105 in the XL group and 23 in the AR cohort. The most frequent causative agents were *S. aureus* (n = 13), *Serratia* (n = 11), *Klebsiella* (n = 7), *Aspergillus* (n = 6) and *Burkholderia* (n = 4). There were 15 presumed fungal infections based on the response to antifungal therapy and/or suggestive laboratory tests (eg, detecting fungal elements on a biopsy). The remaining serious infections were presumed to be bacterial in origin.

The most common serious infectious syndromes (not all of which had etiologic agents identified) were lymphadenitis (n = 29), skin abscess (n = 19), bacterial/presumed bacterial pneumonia/lung abscess (n = 20), fungal/presumed fungal pneumonia/lung abscess (n = 18) and liver abscess (n = 13). Other serious infections included bone abscess/osteomyelitis (n = 5), sepsis (n = 4; usually because of *Burkholderia*), abdominal abscess (n = 4), sinusitis (n = 3) and serious intravenous line infections (n = 2). Liver abscesses (caused by *S. aureus* in all cases where a pathogen was identified) were particularly burdensome in our patient population. Of the 7 patients with 13 liver abscesses at our institution, a total of 67 months was spent hospitalized or on intravenous antibiotics at home; there were 18 separate admissions and multiple surgical procedures (mainly drainage procedures) for these 13 infectious episodes. There was a nonsignificant trend toward more liver abscesses in the patients with XL inheritance (31%) than those with AR inheritance (14%, *P* = 0.37; 6 of our 7 patients with liver abscesses had XL inheritance).

Two patients with CGD colitis had hospital admissions for *Clostridium difficile* diarrhea and were thus counted among the serious infections, whereas 3 other patients had episodes of *C. difficile* diarrhea requiring outpatient therapy or oral therapy while hospitalized for another reason and were not counted as serious infections. There were also 2 episodes each of brain abscess (because of *Aspergillus*) and urinary tract infection/pyelonephritis. The serous infection rate in our series was 0.62 per patient-year of follow-up, with a trend toward more severe infections per year in the XL group (0.71) than in the AR group (0.44; *P* > 0.05)

Fifteen patients (55%) had a range of dermatologic conditions, including frequent, mild skin infections (n = 6), severe cystic acne (n = 6), aphthous ulcers/angular cheilitis/herpes gingivostomatitis/herpangina (n = 5), anal fissures (n = 3), herpes zoster (n = 2), warts (n = 2), eczema/dry skin (n = 2), paronychia (n = 2), ichthyosis vulgaris (n = 1), alopecia areata (n = 1), hordeolum (n = 1), eyelid papilloma (n = 1) and molluscum (n = 1). One patient, the product of a consanguineous marriage, developed a glioblastoma multiforme of the thalamus.^32^

Thirteen patients experienced noninfectious granulomatous complications including colitis (n = 10), eye findings (n = 4; chorioretinitis, retinitis pigmentosa, myopia/astigmatism and episcleritis), liver/lung/skin granulomas (n = 4), hydronephrosis (n = 3), eosinophilic cystitis^30^ (n = 2) and 1 each vitiligo and gastric outlet obstruction. Some patients had >1 noninfectious, granulomatous manifestation of illness. There was no significant difference in the incidence of noninfectious complications between patients with XL (11/19) as opposed to AR (3/8) modes of inheritance (*P* = 0.4).

### Height and Weight

**FIGURE 1.**
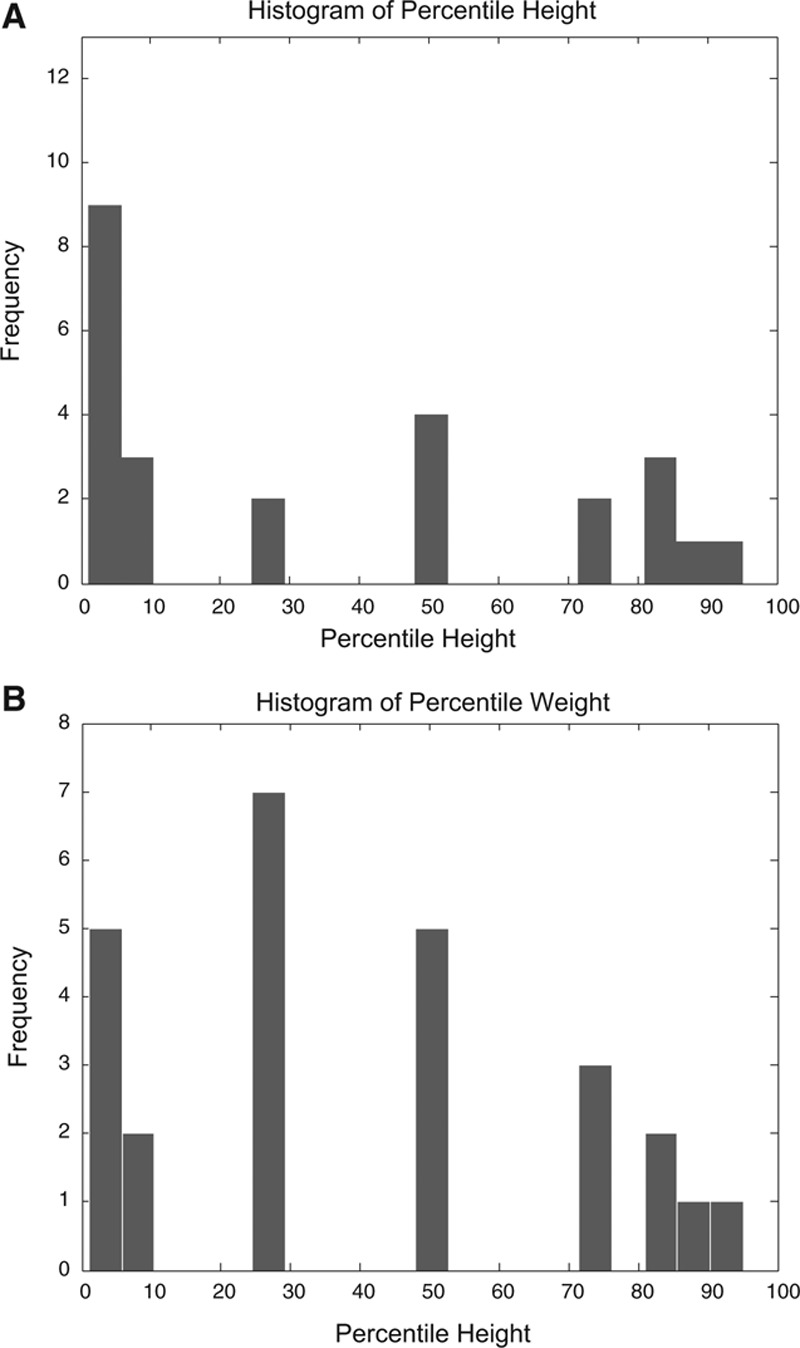
A, Histogram of percentile heights. B, Histogram of percentile weights.

Heights and weights recorded at the time of the last clinic visit revealed that heights of 9 patients were <5th percentile and 12 patients were <10th percentile, whereas 5 patients were <5th percentile and 7 patients were <10th percentile. See Figure 1. There were no significant differences in height or weight whether inheritance was XL or AR recessive (*P* > 0.05; data not shown).

### Treatment

Subcutaneous interferon-γ was Food and Drug Administration approved in 1991, although some of our patients began therapy with this agent in 1990. Twenty-four of 27 patients were consistently maintained on the thrice-weekly administration of subcutaneous interferon-γ (unless they suffered from severe colitis) and daily oral prophylaxis with trimethoprim-sulfamethoxazole and an azole (usually itraconazole since 2003^17^) beginning shortly after the diagnosis was made. One patient was not compliant with therapy and was lost to follow-up. A second patient left our care in 2008, obtained a stem-cell transplant at another institution while healthy in 2010 and is alive at the time of this writing, although we censored his data after he left our institution.

Many of our patients develop fever after the administration of the interferon, which we prepare parents to expect and they learn to tolerate. We generally train parents to administer the interferon subcutaneously and arrange for insurance coverage and home delivery during the child’s initial hospitalization at the time the diagnosis is established. Our patients have found the Compass (Comprehensive Personalized Patient Prescription Advocacy & Support Services) Program (Vidara Therapeutics/Horizon Pharma, Deerfield, IL) helpful regarding medication access, financial assistance, insurance issues, patient information and needle disposal.

### Mortality

**FIGURE 2.**
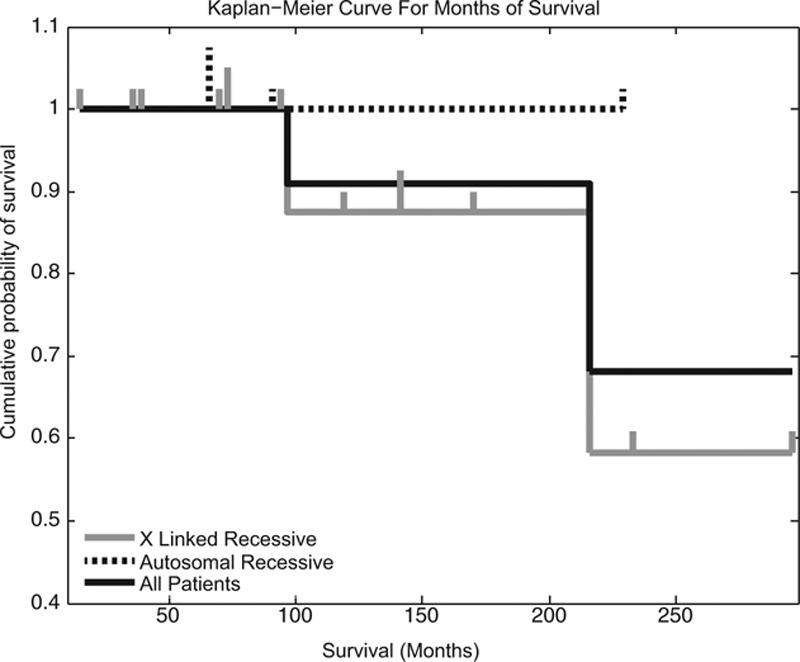
Kaplan–Meier curve for months of survival.

Twenty-three patients were alive as of November 2013; 1 was lost to follow-up. One patient died from intractable pneumonia, sepsis and cerebral edema of unknown etiology in 1992. One patient was referred for stem-cell transplant and died of measles inclusion body encephalitis in 2003.^29^ A third patient died from *Burkholderia* sepsis in 2012. All 3 deaths were in patients with XL inheritance. See Figure 2 for Kaplan–Meier estimates of survival by month. Overall mortality in our compliant patients followed through November 2013 was thus 11.5% over 10.1 years of (average) follow-up per patient; if the patient lost to follow-up is assumed to have died, and our mortality is 15.3%.

## DISCUSSION

Our study represents a large, single-center US experience with CGD. Twenty-four of 27 patients were fairly consistently maintained on thrice-weekly subcutaneous interferon-γ and daily oral prophylactic trimethoprim-sulfamethoxazole and an azole while under our care. In the United States and Europe, about 70% of patients with CGD are on prophylactic antibiotics (usually trimethoprim-sulfamethoxazole), about 50%–70% are on prophylactic antifungals, but only about 33%–35% are on interferon-γ, likely because of intolerance or lack of access (both of which have not been a problem in our patients).^5,23^

Age at diagnosis decreased with time, likely because of better diagnostic testing and increased disease awareness, as has been reported previously.^13^ Our sex ratio and ethnic mix reflect what is known about the 2 forms of the disease and the ethnic mix of patients at our institution. As reported by others, we had several patients below the 5th percentile for height and weight.^5,11,13,14^ (Fig. 1).

Clinical manifestations in our population were similar to what has been reported previously in large studies from developed countries.^1,3–16^ Our serious infection rate of 0.62 per patient-year compares favorably with what has been reported in the literature (0.26–0.7 per patient-year).^10–12^ Although there may be some subjectivity to what is counted as a serious infection, we defined these before we reviewed the patients’ charts. Our definition matches the one used in previous studies,^10–12^ and the data were reviewed by at least 2 physicians who concurred on the assignment in every case. Ten of 27 (37%) of our patients had colitis, very similar to the 33% rate previously reported in a large retrospective analysis of colitis in patients with CGD.^33^

Twenty-two of 27 patients are known to be alive after 3276 patient-months of follow-up without having undergone stem-cell transplantation. Excluding the patient lost to follow-up, our mortality of 11.5% over 10.1 years of follow-up per compliant patient compares favorably with what has been reported in a large series from developed countries since the year 2000 (9%–23 % over 7–16 years of follow-up per patient), the majority of whom also did not undergo stem-cell transplantation.^10–12,22^ There were no deaths among our patients with AR inheritance (see Fig. 2). Even if we assume that the patient lost to follow-up is no longer alive, our mortality still falls well within the ranges reported previously (15.3%).

There are 3 asymptomatic patients in our cohort (ages 9, 12, and 13 years at last follow-up) who were only diagnosed because of a sibling with disease; these children lower the mortality and rate of serious infections as noted previously.^13^ However, because they all have XL disease, they do not affect the morbidity or mortality of the AR cohort.

Gene therapy and bone marrow/stem-cell/cord blood transplantation [hematopoietic stem-cell transplantation (HSCT)] hold out the promise of cure and have been reviewed recently.^21,23^ Gene therapy has not yet resulted in long-term or high-level engraftment or sustained clinical benefits, likely because the infused genetically corrected cells lack a selection advantage in vivo and lack of an optimally defined conditioning regimen.^21^ HSCT is more promising in that the conditioning regimens are better defined, and recent data are generally more encouraging^5,8,9,22–27^; however, follow-ups are shorter (eg, 5–8 years), up to 40% of survivors can remain on chronic medications^22,25,27^ and there is morbidity associated with HSCT (eg, rejection, graft versus host disease, posttransplant lymphoproliferative disease and infection^4,5,8,9,11,12,22–27^), as we experienced in our patient who developed measles inclusion body encephalitis after HSCT.^29^ When reported patients who do and do not undergo HSCT are compared, mortality is not dramatically different (13% over 6 patient-years of follow-up for those who underwent HSCT vs. 8.8% over 8.4 years of patient follow-up for those who did not in one study^22^ and 37% over 21 years vs. 7% over 7.7 years in another study^9^), although everyone who did not undergo HSCT was on chronic medications (vs. up to 40% of those who did^22,25,27^), and quality of life in children whose HSCT was successful is comparable with that of healthy children.^34^ Recently, 21-month survival of 93% with an event-free survival of 89% was reported with reduced-intensity conditioning, human leukocyte antigen–matched donors and therapeutic drug monitoring of busulfan.^27^

Our experience demonstrates that reasonable outcomes can be expected with medical therapy of CGD alone, which includes interferon-γ, trimethoprim-sulfamethoxazole and an azole. We anticipate continued therapeutic advances that will guarantee cure for all children with CGD in the foreseeable future.

## ACKNOWLEDGMENTS

The authors thank Dr Stanford T. Shulman for helpful discussions.
